# The Evaluation of Geroprotective Effects of Selected Flavonoids in *Drosophila melanogaster* and *Caenorhabditis elegans*

**DOI:** 10.3389/fphar.2017.00884

**Published:** 2017-12-07

**Authors:** Ekaterina Lashmanova, Nadezhda Zemskaya, Ekaterina Proshkina, Anna Kudryavtseva, Marina Volosnikova, Elena Marusich, Sergey Leonov, Alex Zhavoronkov, Alexey Moskalev

**Affiliations:** ^1^Department of Biological and Medical Physics, Moscow Institute of Physics and Technology, Dolgoprudny, Russia; ^2^Institute of Biology, Komi Scientific Center of Ural Branch of RAS, Syktyvkar, Russia; ^3^Department of Ecology, Syktyvkar State University, Syktyvkar, Russia; ^4^Engelhardt Institute of Molecular Biology, Russian Academy of Sciences, Moscow, Russia; ^5^Insilico Medicine, Inc., Johns Hopkins University, Baltimore, MD, United States

**Keywords:** lifespan, *D. melanogaster*, *C. elegans*, chrysin, luteolin, naringin

## Abstract

Flavonoids is an intensively studied group of natural compounds with antioxidant, antineoplastic, antihyperglycemic, cardioprotective, and neuroprotective properties. The present study intends to investigate the geroprotective action of three selected flavonoids (naringin, luteolin, chrysin) in two model organisms, *Caenorhabditis elegans* and *Drosophila melanogaster*. Luteolin and chrysin were shown to improve lifespan parameters when administered to both model organisms. The observed positive effects of these flavonoids in *D. melanogaster* were limited to females and were not associated with reduced fecundity or locomotor impairment. The life-extending effects of flavonoids were observed in N2 wild-type worms but absent in *aak-2(gt33)* mutants implying that these effects can be associated with AMP-activated protein kinase activity. Naringin improved lifespan parameters of *C. elegans*, but had no effect on *D. melanogaster* females; in some cases, naringin was found to decrease the lifespan of males. Compared to chrysin and luteolin, however, naringin more effectively activates Nrf2 target genes (particularly, *GstD1*) under oxidative stress. Then we compared molecular mechanisms of studied compounds and a well-known geroprotector rapamycin, using software tool GeroScope. There are no transcriptomic data on luteolin or chrysin provided by LINCS Project database. The bioinformatics comparison of transcriptomics data for A549 and MCF7 human cell lines treated with rapamycin or naringin revealed that these compounds share just a few common signaling pathways and quite distinct in their geroprotective action. Thus, based on *C. elegans* effects of naringin, luteolin, chrysin on lifespan we have revealed new potential geroprotectors.

## Introduction

Experiments conducted on various model organisms have shown that it is possible to pharmacologically modify the activity of longevity-associated signaling pathways. Flavonoids are a group of naturally occurring biologically active compounds (Zern and Fernandez, 2005; Vauzour et al., 2008; Kumar and Pandey, 2013; Brodowska, 2017). The geroprotective effects of different flavonoids on model organisms have been explored in numerous studies, the majority of which use *Caenorhabditis elegans* as a model organism to investigate the effects of flavanols, flavonols, and plant extracts (Pallauf et al., 2017). However, this area of research has not been without contention. Experiments involving the use of one compound have produced inconsistent or controversial results when performed on multiple model organisms. For example, quercetin was found to increase the lifespan of *Saccharomyces cerevisiae* (Belinha et al., 2007) and *C. elegans* (Kampkotter et al., 2008; Saul et al., 2008; Pietsch et al., 2009), but had no effect on mice (Jones and Hughes, 1982; Spindler et al., 2013). Those studies highlights the importance of the reproducibility of a compound’s geroprotective effects among different model organisms, particularly given that longevity-associated signaling pathways are highly evolutionarily conservative (Moskalev et al., 2016). For example, the increased lifespan of multiple organisms was observed for the anti-inflammatory drug ibuprofen (He et al., 2014) and the immunosuppressant rapamycin (Harrison et al., 2009; Bjedov et al., 2010; Robida-Stubbs et al., 2012).

Recently, it was reported that the flavone baicalein increases the lifespan and stress resistance of *C. elegans* (Havermann et al., 2013, 2016). Flavones are a subgroup of flavonoids with antineoplastic (Seelinger et al., 2008), anti-inflammatory (Ahad et al., 2014), and antihyperglycemic properties (Vinayagam and Xu, 2015). To further investigate the geroprotective activity of this subgroup, we first verified their effects on *C. elegans* lifespan via two other flavones, luteolin (3′,4′,5,7-tetrahydroxyflavone), and chrysin (5,7-dihydroxyflavone). Luteolin is abundant in the human diet, contained in foods like broccoli, carrots, parsley, and bird’s eye chilies, among others (Miean and Mohamed, 2001). Chrysin is less prevalent in the human diet but can be found in honey and propolis (Gambelunghe et al., 2003). The impact of those flavones on resistance of two model organisms to different stress conditions was also investigated. Subsequently, we comprehensibly examined *Drosophila melanogaster* as a model organism, studying various physiological parameters (fecundity, spontaneous activity) in addition to lifespan assays. To explain the observed effects, we also investigated the molecular mechanisms of flavonoids’ action using RT-PCR method as well as *C. elegans* mutant and *D. melanogaster* transgenic strains. It is known that flavonoids can influence evolutionary conserved signaling pathways. For example, they can induce (AMP)-activated protein kinase (AMPK) (Hwang et al., 2011; Pu et al., 2012; Shao et al., 2012). Thus we performed experiments with TG38 *aak-2(gt33) C. elegans* strain, which has a deletion in the *aak2* gene. This gene codes one out of two homologs of alpha catalytic subunits of mammalian AMPK (Lee et al., 2008). In *D. melanogaster* the influence of compounds on *AMPK* gene expression was measured. Using *D. melanogaster* model we also investigated the effects of selected flavonoids on Keap1/Nrf2 signaling pathway by measuring the expression values of certain genes coding proteins of this pathway and expression level of *GstD1-GFP* reporter that is Nrf2 target under both stress and non-stress conditions. We have extended our study to include naringin, a flavanone-7-*O*-glycoside found in citrus fruits and juices, since naringenin has recently been shown to have hormetic effects on *D. melanogaster* longevity (Chattopadhyay et al., 2016) and glycosidic forms of flavonols are also known to be bioactive (Lee et al., 2015). As the hormetic effects are usually considered as the ability to induce cell stress defense program in the absence of stress, the effects of selected flavonoids on expression of some stress response genes was studied.

## Materials and Methods

### *C. elegans* Lifespan Assay

In this experiment, two *C. elegans* strains were used; the Bristol strain N2, kindly provided by Yelena Budovskaya (University of Amsterdam), and the TG38 *aak-2(gt33)* strain, kindly provided by Alexander Mironov (Engelhardt Institute of Molecular Biology, Moscow). The experiments were performed in liquid culture at 20°C according to a slightly modified version of the protocol set forth by Solis and Petrascheck (2011). In brief, worms were synchronized with a bleach solution. After hatching, they were placed in 96-well plates with an S-complete solution and *Escherichia coli* OP50 as a food source. To prevent the production of progeny, a 0.12 mM solution of 5-fluoro-2′-deoxyuridine (FUDR) (Sigma) was added to *C. elegans* approximately 45 h after adding the bacteria to L1 worms. Amphotericin B was used at a final concentration of 0.1 μg/mL. To prevent starvation, 5 μl of OP50 (2 × 10^9^ CFU/ml) was added to each well on the 5th, 12th, and 19th day of the worms’ adulthood. The DMSO stock solutions of luteolin (Sigma), chrysin (Acros Organics) and naringin (Acros Organics) were added to N2 nematodes at final concentrations 0.5, 1, 5, 10, and 100 μM. The experiments on the *aak-2(gt33)* mutant strain were performed using the above concentrations of flavonoids, demonstrating the effect of flavonoids on wild-type worms. This mutant strain has a deletion in the *aak2* gene coding, leaving one out of two alpha catalytic subunits of AMPK (Lee et al., 2008). For each experimental group in each experiment 45–110 nematodes were used with the total amount of worms in all performed experiments being no less than 170 for N2 Bristol strain and 110 for TG38 *aak-2(gt33)* strain. The number of dead worms was counted three times per week. Wells with less than five or more than 15 worms were eliminated to control for the effects of varied food availability. Vibratory perturbation and light exposure were used to induce movement and improve the accuracy of the count. It should be mentioned that in some cases, sediment was observed. The experiment was replicated three times. Some flavonoid concentrations were tested only twice. The statistical analysis was performed for each experiment and for the combined data of all experiments. The survival functions were counted using the Kaplan–Meier method and plotted as survival curves (Kaplan and Meier, 1992). The mean, median, minimum and maximum lifespans as well as the age of 90% mortality were calculated. The statistical significance of differences in survival data was estimated using the non-parametric Kolmogorov–Smirnov (Fleming et al., 1980), Cox–Mantel (Mantel, 1966) and Gehan-Breslow-Wilcoxon (Breslow, 1970) tests. The statistical significance of differences in maximum lifespan (the age of 90% mortality) was calculated using Wang–Allison test (Wang et al., 2004). The aging process was also assessed by calculating age-dependent (α) and initial (R_0_) mortality rates in the Gompertz equation (μ(x) = R_0_ e^αx^) and mortality rate doubling time (MRDT) using the formula MRDT = ln2/α (Finch, 1990). All calculations were performed using Statistica 6.1 (StatSoft, United States) and software environment for statistical computing and graphics R 2.15.1. The effects were considered to be significant if they were observed in at least two out of three experiments in addition to the data from the three experiments combined.

### *C. elegans* Stress Resistance Assay

The experiments to assess stress resistance were performed on the 5th day of flavonoid treatment. In experiments regarding oxidative stress, 100 mM paraquat (Methyl Viologen, Acros Organics) was added to nematodes. In experiments testing heat shock, the worms were heated to 33°C. The number of dead worms was counted after 24 h. For each type of stress, the experiment was performed in triplicates. For each experimental group in each experiment 35–117 nematodes were used with the total amount in all performed experiments being no less than 170 for each variant. The data of three experiments was combined. The statistical comparison of the percentage of dead animals for combined data was made using the Fisher ϕ-test (Fisher, 1954).

### *D. melanogaster* Lifespan Assay

The wild-type Canton-S *D. melanogaster* (Bloomington Stock Center, United States) were separated by sex and housed in 25 mm × 95 mm vials (30 flies per vial) with sugar-yeast medium of the following composition per 1 L: dry yeast—8 g, agar—7 g, sugar—30 g, semolina—30 g, propionic acid—8 drops (Ashburner, 1989). For each experimental group 100–150 flies were used. The flies were kept at 25°C with a 12-h light/dark cycle. The DMSO stock solutions of the studied compounds were dissolved in the yeast paste at final concentrations or 0.3, 0.5, and 1 μM. These concentrations were chosen as they are considered physiological (Kanazawa, 2011). This mixture was applied to the surface of the nutrient medium starting on the first day of adulthood. The flies were transferred into new bottles twice per week. The number of dead flies was counted no less than three times per week. The experiment was replicated three times. The same statistical analyses as for worms was performed.

### *D. melanogaster* Stress Resistance Assay

The experiments on stress resistance were performed on the 10th day of flavonoid treatment. For each experimental group 100–150 flies were used. In experiments testing oxidative stress, flies were moved into vials with filter paper soaked in a 20 mM paraquat solution (Methyl Viologen, Sigma) in 5% sucrose (0.2 mL per vial) after 2 h of starvation. The flies were starved by transfer into vials with a filter paper saturated with water instead of the nutrient medium. In experiments regarding heat shock stress, the vials containing the flies and the nutrient medium were warmed to 35°C. The number of dead flies was counted twice a day. For each type of stress, the experiment was performed in triplicates. The data of three experiments was combined. The significance of differences between groups for combined data was evaluated with the Fisher ϕ-test (Fisher, 1954).

### *D. melanogaster* Fecundity Assay

The fecundity of females was assessed once per week by placing three females and three males of the same age into vials with fresh nutrient colored with activated carbon and allowing 24 h for egg laying. After 24 h had passed, the number of eggs per female was counted. For each experimental variant, 50 fertile females were used. The males were replaced with young flies once per month. The statistical significance between the cumulative curves was evaluated with the χ^2^ test using the program Statistica 6.1 (StatSoft, United States) (Fisher, 1954).

### *D. melanogaster* Spontaneous Activity Assay

For each experimental variant, 30 flies were selected (10 flies per vial). Males and females were analyzed separately. The flies were kept in standard conditions and transferred to new media twice per week. The spontaneous activity was tested for 24 h each week with the hardware-software complex “Drosophila Population Monitor” (TriKinetics, Inc., United States). The statistical analyses were performed with the χ^2^ test using the program Statistica 6.1 (StatSoft, United States) (Fisher, 1954).

### RT-PCR

In this test, only 0.3 and 1.0 μM concentrations of the selected compounds were studied. On the 10th day of flavonoid consumption, the flies were homogenized. RNA was extracted using QIAzol Lysis Reagent (Qiagen, Netherlands) with subsequent isopropanol precipitation. RNA was purified with the DNase I, Amplification Grade kit (Life Technologies, Breda, the Netherlands) in accordance with the manufacturer’s protocol. The synthesis of cDNA was performed using Revert Aid reverse transcriptase as recommended by Fermentas. Real-time PCR was carried out on the 7500 Real-Time PCR System (Applied Biosystems, United States) using the following procedure: (1) denaturation for 10 min at 95°C, (2) denaturation for 15 s at 95°C, (3) annealing for 30 s at 60°C, (4) elongation for 30 s at 60°C. Steps 2–4 were repeated 50 times.

*EF1*α and β-*Tubulin* were used for expression normalization as they were the most stable among the four tested genes (*Actin*, *RpL32*, *EF1*α, β-*Tubulin*). The relative expression of the studied genes was calculated as described earlier (Zhikrevetskaya et al., 2015). The primer sequences for studied genes are presented in Supplementary Table 1.

The differences between relative expression values were counted as significant only if they were statistically significant according to the Student’s *t*-test and at least a twofold change in expression of the studied gene was considered due to biological variations in expression levels of reference genes (Log2FC > 1).

### Quantification of GFP Reporter Gene Expression

In this experiment, the *D. melanogaster* transgenic line with *GstD1-GFP* reporter was used. The line was kindly provided by Dr. Tower (University of Southern California, Los Angeles, CA, United States).

To measure *GstD1-GFP* reporter expression, flies were anesthetized and photographed using a fluorescent microscope “MICMED-2 var.11” (LOMO, Russia) and video systems based on the Olympus C7070 digital camera (Olympus, Japan) on the 10th and 30th days of flavonoid consumption. The corrected total cell fluorescence (CTCF) was calculated using ImageJ software (National Institutes of Health, United States) as described elsewhere (Cali et al., 2015). The same assay was performed on flies treated for 12 h with 20 mM paraquat (Methyl Viologen, Sigma) in 5% sucrose (0.2 mL per vial). For each experimental group 10 flies were used.

### Pathway-Level Similarity Analysis

Signaling pathway analysis is a common approach to gain insight from large-scale transcriptomic and proteomic data. To obtain signaling pathway activation scores (PASs), we utilized the *in silico* Pathway Activation Network Decomposition Analysis (iPANDA), which we applied to large-scale transcriptomic datasets as a method for biomarker development (Ozerov et al., 2016). In contrast to other methods, iPANDA generates PASs by using precalculated gene coexpression data in combination with gene importance factors quantified according to the degree of differential gene expression and pathway topology decomposition.

To obtain signaling PASs, we utilized transcriptional response data provided by LINCS Project^1^. We chose gene expression samples of drug-induced transcriptional response from A549 and MCF7 cell lines. The collection of signaling pathways is obtained from the SABiosciences collection^2^, in which there are 378 signaling pathways. A total of 15,489 compounds were considered. The similarity between two compounds was measured as a percent of commonly up- or down-regulated pathways. The pathway-level similarity analysis was performed only for naringin as there is no transcriptional response data for chrysin and luteolin.

## Results

### The Effects of Studied Flavonoids on *C. elegans* Lifespan

**FIGURE 1:**
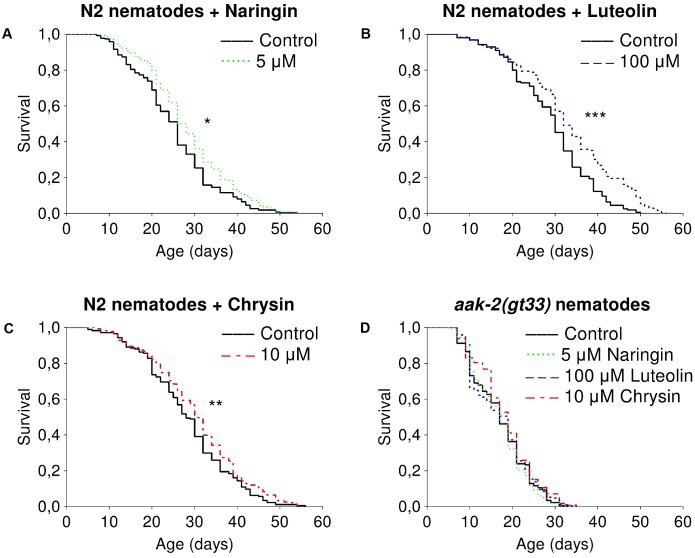
Survivorship curves of *Caenorhabditis elegans* N2 Bristol **(A–C)** and TG38 *aak-2(gt33)* **(D)** strains treated with flavonoids (combined data of two-three independent replicates); ^∗^*p* < 0.05, ^∗∗^*p* < 0.01, ^∗∗∗^*p* < 0.001, Kolmogorov–Smirnov test.

The studied flavonoids had positive effects on median lifespan of nematodes in certain concentrations (Supplementary Table 2). The most pronounced effects of naringin were observed for concentrations of 5 μM (**Figure 1A**). The addition of naringin in this concentration increased the median lifespan of worms by 7.7–15.4% (*p* < 0.05) in all performed experiments. The median and maximum lifespan were increased by 6.3–6.7 and 16.6–25% (*p* < 0.05), respectively, after the addition of luteolin at a concentration of 100 μM (**Figure 1B**). Chrysin, in a concentration of 10 μM, significantly increased the median lifespan by 6.7–30.7% (*p* < 0.05) in two experiments out of three (**Figure 1C**). However, the pro-longevity action of flavonoids was absent among nematodes with the *aak2* mutation (**Figure 1D**). These data suggest that the effects were at least partly associated with AMPK (Supplementary Table 3).

### The Effects of Studied Flavonoids on *C. elegans* Stress Resistance

**FIGURE 2:**
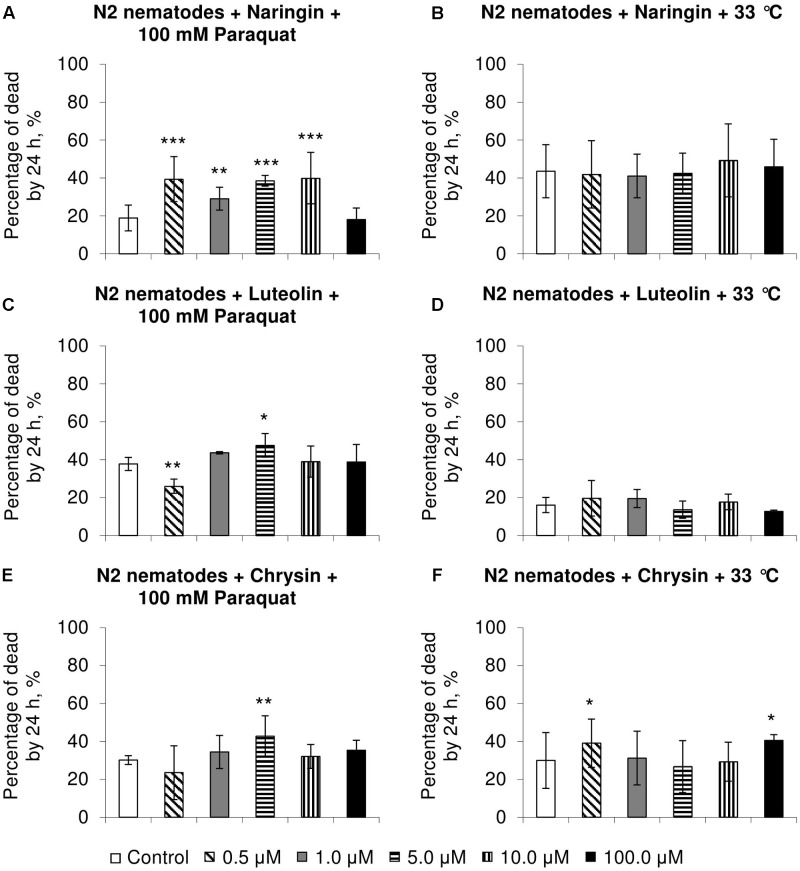
Resistance of *Caenorhabditis elegans* to oxidative stress (100 mM paraquat) **(A,C,E)** and heat shock (33°C) **(B,D,F)** after treatment with naringin **(A,B)**, luteolin **(C,D)**, and chrysin **(E,F)** (combined data of three independent replicates); ^∗^*p* < 0.05, ^∗∗^*p* < 0.01, ^∗∗∗^*p* < 0.001, Fisher’s exact test.

Naringin did not affect heat shock resistance in *C. elegans* (**Figure 2B**) but increased the number of dead nematodes by 54–111% (*p* < 0.01) in experiments on oxidative stress (**Figure 2A**). In most cases, chrysin and luteolin did not significantly affect, or in some cases decreased, the survival rate of nematodes under stress conditions (*p* < 0.05) (**Figures 2C–F**).

### The Effects of Studied Flavonoids on *D. melanogaster* Lifespan

**FIGURE 3:**
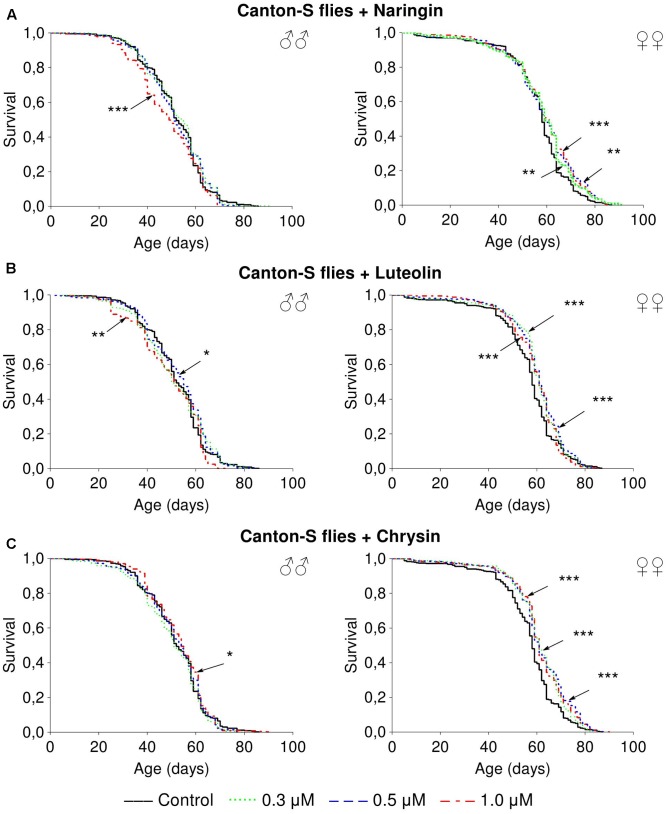
Survivorship curves of *Drosophila melanogaster* males (♂♂) and females (♀♀) treated with naringin **(A)**, luteolin **(B)**, and chrisin **(C)** (combined data of three independent replications); ^∗^*p* < 0.05, ^∗∗^*p* < 0.01, ^∗∗∗^*p* < 0.01, Kolmogorov–Smirnov test.

Naringin had no significant effect on lifespan parameters of females, but in high concentration (1 μM) decreased the median lifespan of males by 6.5–20% (*p* < 0.05) in most cases (Supplementary Table 4 and **Figure 3A**). The addition of luteolin and chrysin, in all studied concentrations, increased the median lifespan of females by 1.8–12.1% (*p* < 0.01) and the maximum lifespan by 6.2–21.9% (*p* < 0.05) in most cases (Supplementary Tables 5, 6). However, no significant and stable effects were observed for males (**Figures 3B,C**).

### The Effects of Studied Flavonoids on *D. melanogaster* Stress Resistance

**FIGURE 4:**
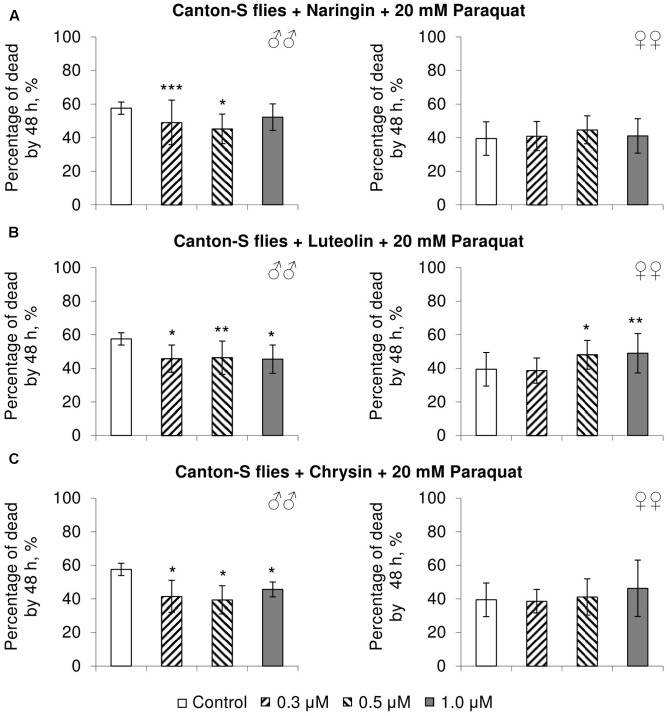
Resistance of *Drosophila melanogaster* males (♂♂) and females (♀♀) to oxidative stress (20 mM paraquat) after treatment with naringin **(A)**, luteolin **(B)**, and chrysin **(C)** (combined data of three independent replicates); ^∗^*p* < 0.05, ^∗∗^*p* < 0.01, ^∗∗∗^*p* < 0.01, Fisher’s exact test.

**FIGURE 5:**
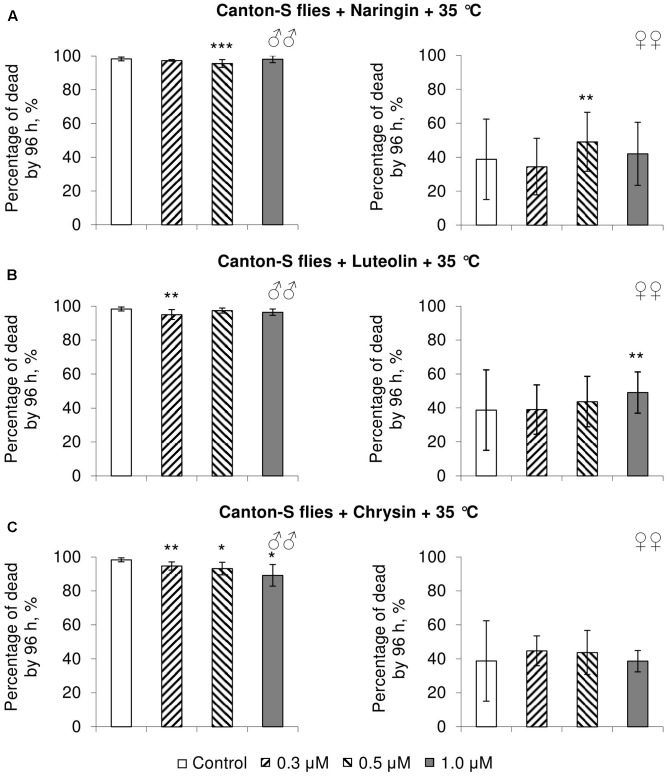
Resistance of *Drosophila melanogaster* males (♂♂) and females (♀♀) to heat shock (35°C) after treatment with naringin **(A)**, luteolin **(B)**, and chrysin **(C)** (combined data of three independent replicates); ^∗^*p* < 0.05, ^∗∗^*p* < 0.01, ^∗∗∗^*p* < 0.01, Fisher’s exact test.

**FIGURE 6:**
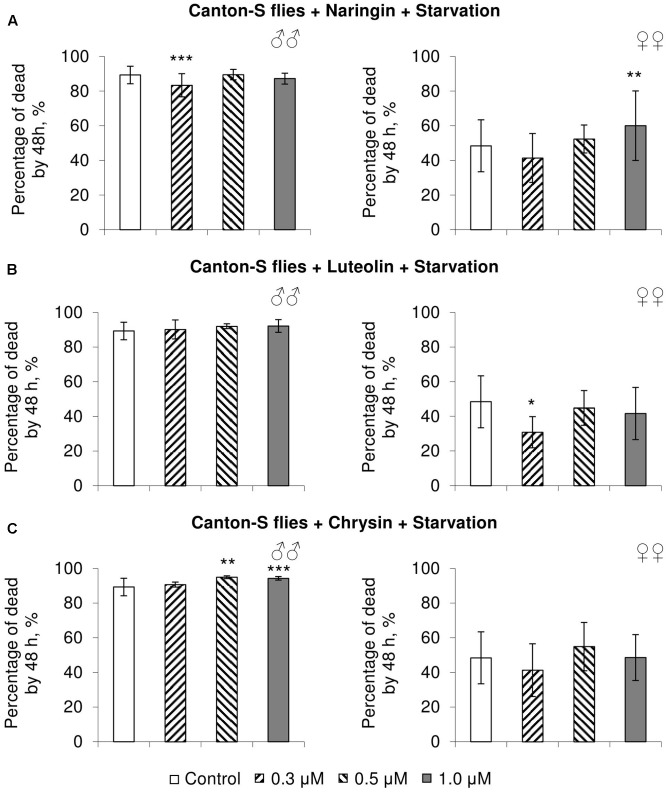
Resistance of *Drosophila melanogaster* males (♂♂) and females (♀♀) to starvation after treatment with naringin **(A)**, luteolin **(B)**, and chrysin **(C)** (combined data of three independent replicates); ^∗^*p* < 0.05, ^∗∗^*p* < 0.01, ^∗∗∗^*p* < 0.01, Fisher’s exact test.

In most cases, naringin either did not or negatively affected the stress resistance of females (*p* < 0.05) (**Figures 4A**, **5A**, **6A**). In the lowest concentration (0.3 μM), luteolin improved starvation resistance in females (**Figure 6B**). Other studied concentrations, however, were found to diminish their resistance to both paraquat and heat shock (*p* < 0.05) (**Figures 4B**, **5B**). Chrysin had no effects on stress resistance of females (**Figures 4C**, **5C**, **6C**).

In most concentrations, all three studied compounds were found to reduce the number of dead males by 14.8–31.4% after incubating for 48 h with paraquat (*p* < 0.05) (**Figure 4**). The addition of chrysin improved male heat shock resistance by 3.7–9.3% to thermal shock but abated their starvation resistance by 5.6–6.4% (*p* < 0.05) (**Figures 5C**, **6C**). The effects of both naringin and luteolin on heat shock and starvation resistance in males had in most cases no significance (**Figures 5A,B**, **6A,B**).

### The Effects of Studied Flavonoids on Flies’ Fecundity and Locomotor Activity

**FIGURE 7:**
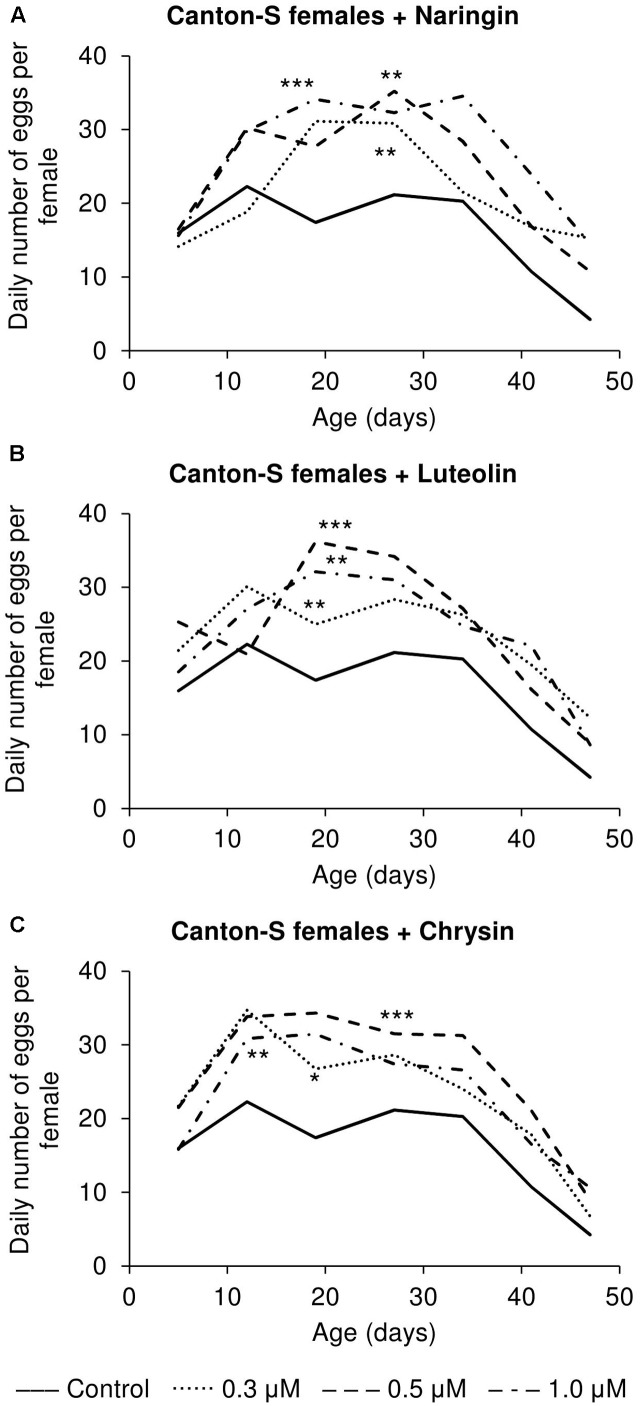
Fecundity activity of *Drosophila melanogaster* females treated with naringin **(A)**, luteolin **(B)**, and chrysin **(C)**; ^∗^*p* < 0.05, ^∗∗^*p* < 0.01, ^∗∗∗^*p* < 0.001, χ^2^ test.

The addition of the studied compounds, in all tested concentrations, was found to increase the number of eggs laid per female (**Figure 7**). However, the most remarkable changes as compared to the control flies were observed in older flies. At that time, the number of eggs laid was found to be increased by up to 3.6 times for naringin, 2.9 times for luteolin, and 2.1 times for chrysin (*p* < 0.05).

**FIGURE 8:**
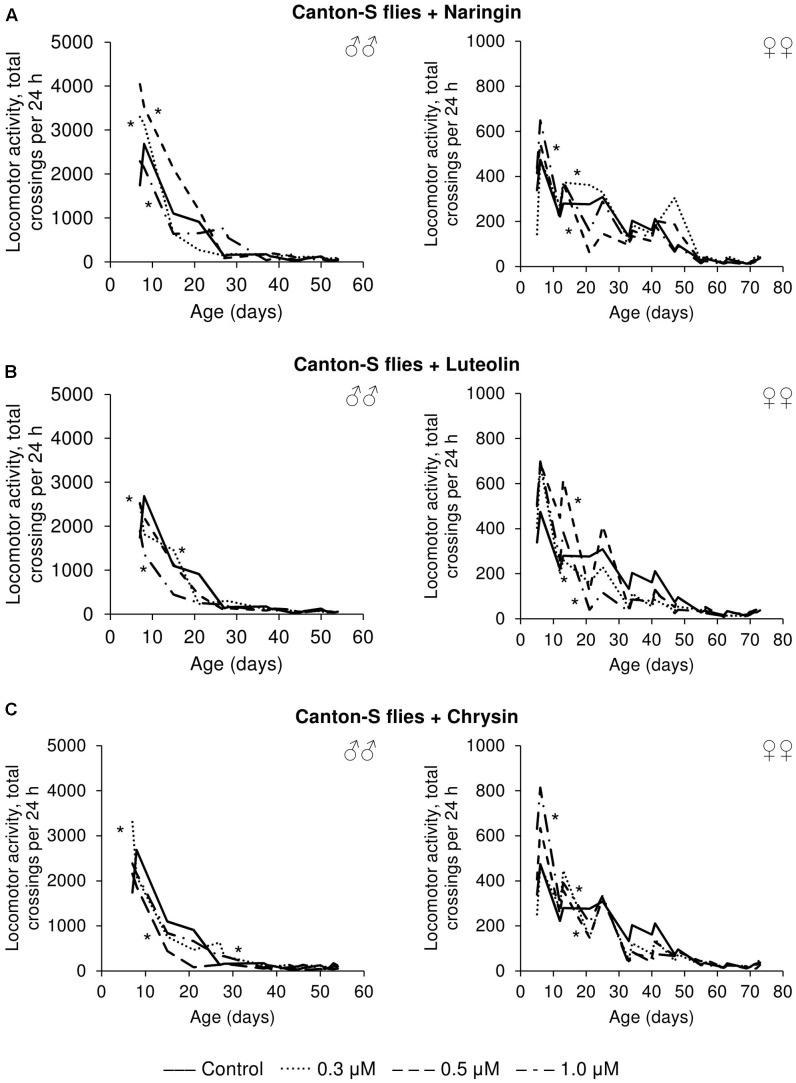
Spontaneous locomotor activity of *Drosophila melanogaster* males (♂♂) and females (♀♀) treated with naringin **(A)**, luteolin **(B)**, and chrysin **(C)**; ^∗^*p* < 0.001, χ^2^ test.

The effects that the tested flavonoids had on spontaneous activity were not so univocal. Even though the statistical analysis revealed changes compared to control flies, it can be concluded that the effects were mainly neutral. While those flavonoids increased the spontaneous activity of both males and females for some time-intervals, they appear to have had deleterious effects over other time periods (**Figure 8**).

### The Effects of Studied Flavonoids on Flies’ Stress Response Genes Expression

**FIGURE 9:**
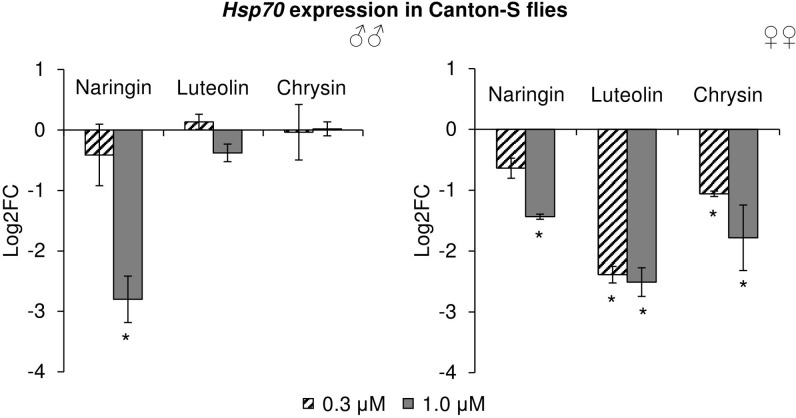
Expression of *Hsp70* gene in *Drosophila melanogaster* males (♂♂) and females (♀♀) treated with flavonoids (combined data of three independent replicates); ^∗^*p* < 0.001, χ^2^ test.

The effects of the studied flavonoids on the expression of stress-response genes were investigated. Among 15 studied genes, only the changes in the *Hsp70* gene can be considered to be significant (**Figure 9**). In females, *Hsp70* expression levels were decreased by 2.1–5.7 times (*p* < 0.05) after the addition of all studied concentrations of chrysin and luteolin. The effects were dose-dependent, with higher flavonoid concentration corresponding to a pronounced decrease in the activity of the *Hsp70* gene. The same effect was observed in females for high concentrations of naringin. In males, 1 μM naringin was found to reduce the expression of the *Hsp70* gene by 6.9 times (*p* < 0.05). Luteolin and chrysin, however, did not significantly affect this gene in males. The results for all other genes were not considered to be significant.

### The Effects of Studied Flavonoids on Keap1/Nrf2 Signaling Pathway in Flies

**FIGURE 10:**
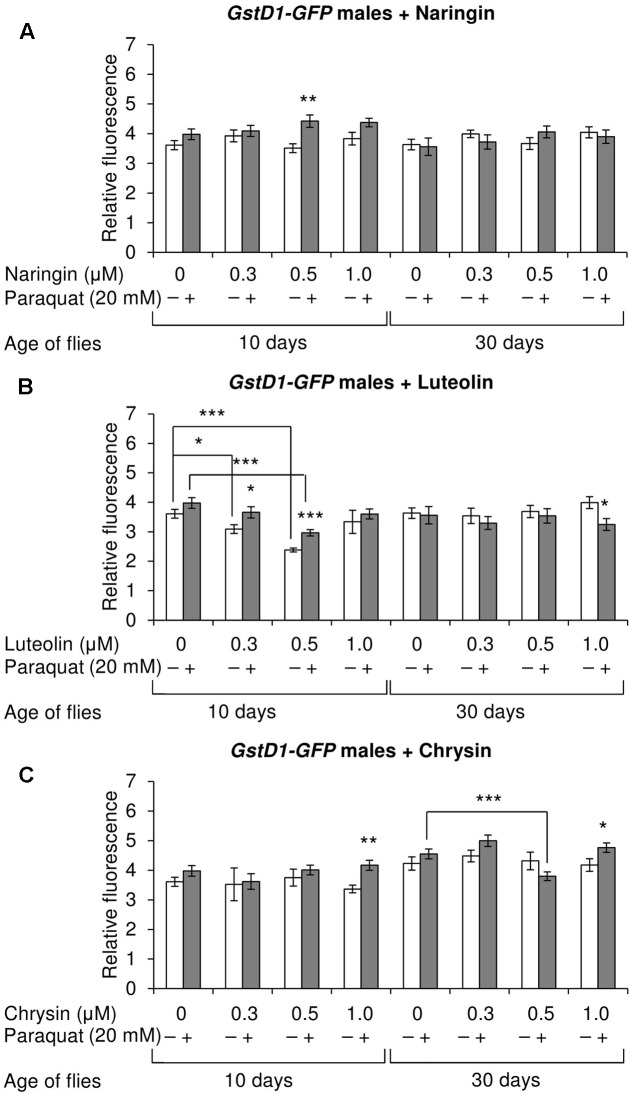
Relative fluorescence of *GstD1-GFP* reporter in *Drosophila melanogaster* males after treatment with naringin **(A)**, luteolin **(B)**, and chrysin **(C)**; ^∗^*p* < 0.05, ^∗∗^*p* < 0.01, ^∗∗∗^*p* < 0.01, χ^2^ test.

**FIGURE 11:**
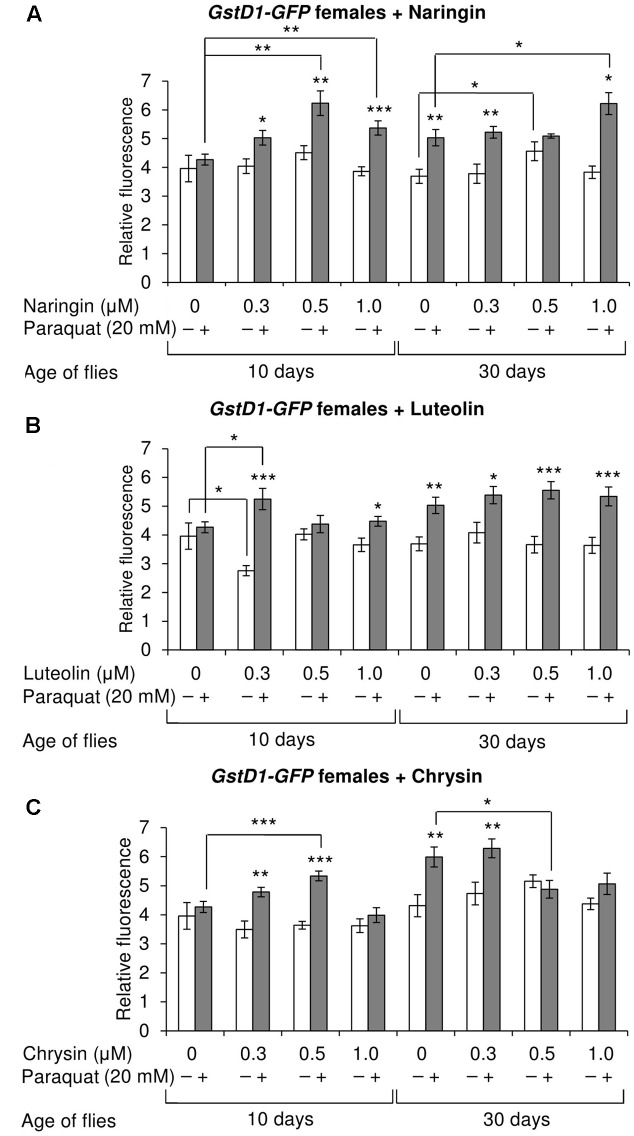
Relative fluorescence of *GstD1-GFP* reporter in *Drosophila melanogaster* females after treatment with naringin **(A)**, luteolin **(B)**, and chrysin **(C)**; ^∗^*p* < 0.05, ^∗∗^*p* < 0.01, ^∗∗∗^*p* < 0.01, χ^2^ test.

The effects of the studied compounds on the Keap1/Nrf2 signaling pathway in flies was tested. No significant effects were observed on the mRNA levels of genes *CncC* (coding the Nrf2 homolog in flies), *Keap1* (coding the CncC inhibitor), or *GclC* (coding the CncC target gene). *GstD1* is another target gene of CncC (Sykiotis and Bohmann, 2008). The flies with the *GstD1-GFP* reporter gene were used to study the effects of flavonoids on the organism’s ability to induce the expression of the Keap1/Nrf2 signaling pathway target gene under both normal and stress conditions. In most cases, chrysin did not affect *GstD1-GFP* reporter expression in either males or females under both normal and stress conditions as compared to the *GstD1-GFP* expression level in either the control group or after 12 h of paraquat treatment (**Figures 10C**, **11C**). In contrast, the expression of this gene was observed to decrease after the addition of luteolin in most cases for both males and females after 10 days of treatment under non-stress conditions (**Figures 10B**, **11B**). No effects were observed for males after the addition of naringin (**Figure 10A**). However, naringin was found to increase *GstD1-GFP* reporter level in females under stress conditions after both 10 and 30 days of treatment and in normal conditions after 30 days (**Figure 11A**). At the same time, it should be noted that 12 h of paraquat treatment on the 10th day of adulthood was not sufficient to significantly increase *GstD1-GFP* reporter level in control flies. In the case of females treated with those flavonoids, the increase was dramatic. Thus, it can be concluded that these flavonoids improve the speed of Nrf2 target genes activation under stress conditions.

### Similarity at Pathway Level

To determine pathway-level similarity, the iPANDA algorithm was applied. For each compound, perturbation PASs were calculated for 378 pathways. The similarity between the pathway activation signatures of natural compounds and rapamycin was evaluated by the number of commonly up- and down-regulated pathways between 15,489 compounds. Results of the pathway-level analysis are depicted in Supplementary Tables 7, 8.

The best similarity (rank 348 out of 15,489) was observed between naringin (time of exposure 24 h, concentration 10 μM) and rapamycin (time of exposure 24 h, concentration 0.4 μM) in A549 cell line, but in this case, the amount of commonly regulated pathways was only 88 out of 378 analyzed. The highest amount of commonly regulated pathways between naringin and rapamycin was 229 and was observed in MCF7 cells. However, when comparing to other compounds the rank of naringin is quite low (rank 1306 out of 15,489). Thus, it can be concluded that naringin and rapamycin have quite distinct mechanisms of action at least in A549 and MCF7 cells.

## Discussion

In certain concentrations, the studied compounds improved the lifespan parameters of the *C. elegans* model organism. Luteolin and chrysin also improved the lifespan parameters of *D. melanogaster* females without reducing aging-dependent physiological parameters (fecundity and locomotor activity) but did not affect males. Naringin did not affect the lifespan of females and, at the highest concentration (1 μM), decreased the lifespan parameters of males. Additionally, flavonoids had either no effect or, in high concentrations, decreased the stress resistance of nematodes and flies.

For females, all studied compounds were associated with decreased *Hsp70* gene expression. For males, only naringin was associated with decreased *Hsp70* gene expression. The down-regulation of the *Hsp70* gene is a known effect of flavonoids like quercetin (Tatsuta et al., 2014), fisetin (Kim et al., 2015), and epigallocatechin-3-gallate (Tran et al., 2010). Hsp70 proteins protect cells from various kinds of stress by participating in proteostasis and playing a role in cellular processes such as apoptosis or proliferation via interaction with regulatory proteins (Mayer and Bukau, 2005; Gong and Golic, 2006). *Hsp70* overexpression is associated with increased lifespan in flies (Tatar et al., 1997). On the other hand, it was observed that flies with higher levels of *Hsp70-GFP* reporter die sooner than flies with lower levels (Yang and Tower, 2009). Thus, low levels of *Hsp70* can be a biomarker of younger biological age.

The results of our experiments on the *C. elegans* mutant strain also suggest that the observed positive effects on nematode lifespan were at least partly associated with (AMP)-activated protein kinase (AMPK). Interestingly, according to the literature, the activation of AMPK leads to a decline of *Hsp70* mRNA stability (Wang et al., 2010). For example, the flavonoid quercetin down-regulated *Hsp70* via AMPK activation in HeLa cells (Jung et al., 2010). The ability to activate AMPK was also shown for luteolin (Hwang et al., 2011), chrysin (Shao et al., 2012), and naringin (Pu et al., 2012). AMPK is a protein kinase that controls cellular metabolism by activating some signaling pathways like Nrf2/SKN-1 and FOXO/DAF-16 and inhibiting others like TOR (Salminen and Kaarniranta, 2012). The overexpression of the AMPK gene in nematodes has been found to increase the lifespan of nematodes and flies (Apfeld et al., 2004; Stenesen et al., 2013; Ulgherait et al., 2014). The overexpression of this gene in the *Drosophila* nervous system has been demonstrated to result in greater effects on lifespan in females and was not associated with the decreased fecundity or spontaneous physical activity (Ulgherait et al., 2014). In our experiments, the effects were also more pronounced among *D. melanogaster* females and did not adversely affect fecundity or spontaneous physical activity. However, it is also possible that the more dramatic expression of these effects in females is a result of their higher food consumption (Wong et al., 2009).

According to the literature, another flavone, baicalein, has been found to improve the lifespan and stress resistance of *C. elegans* by activating the Keap1/Nrf2 signaling pathway, which regulates the cell-protection mechanisms (Havermann et al., 2013, 2016). However, chrysin and luteolin can either inhibit or activate this pathway depending on the biological model used in the experiments (Keum and Choi, 2014). For example, luteolin has been shown to inhibit the Keap1/Nrf2 signaling pathway in human hepatoma (HepG2), rat liver epithelial (RL-34), and mouse hepatoma (Hepa1c1c7) cells treated with dioxin (TCDD) (Zhang et al., 2014). The same effect was observed in another study on human lung carcinoma A549 (NSCLC), human breast carcinoma (MCF7), and human colon cancer (Caco2) cell lines (Tang et al., 2011). The effects were associated with a decrease of Nrf2 stability (Tang et al., 2011; Zhang et al., 2014). Similarly, chrysin was found to inhibit this signaling pathway in parental human hepatocellular carcinoma cells (Bel-7402/ADM) (Gao et al., 2013). At the same time, both chrysin and luteolin have been demonstrated to induce Nrf2 activity in hepatocytes isolated from male Sprague–Dawley rats, thus increasing their resistance to oxidative stress (Huang et al., 2013). In our experiments, chrysin and luteolin had no effects on the level of Nrf2 target activation under both stress and non-stress conditions. In some variants, luteolin even decreased the expression of the *GstD1-GFP* reporter. In contrast, naringin increased the level of *GstD1-GFP* reporter under normal and non-stress conditions. Naringin’s ability to activate the Keap1/Nrf2 signaling pathway is consistent with other studies (Gopinath and Sudhandiran, 2012; Chen et al., 2015). However, it is known that the level of Nrf2 target genes does not decrease with age, but the ability to activate under stress conditions does (Rahman et al., 2013). Our data imply that all three studied compounds increase the speed of Keap1/Nrf2 signaling pathway activation under paraquat treatment in flies.

Luteolin and chrysin have two and zero hydroxyl groups, respectively, on the B-ring. According to literature, the ability of flavonols, another subclass of flavonoids, to increase the lifespan of model organisms depends on the number of hydroxyl groups on their B-ring, with more hydroxyl groups being associated with more pronounced effects (Grunz et al., 2012). In our experiments, we observed no drastic differences between the effects of luteolin and chrysin on lifespan parameters of *C. elegans* and *D. melanogaster*. However, the addition of luteolin resulted in a more pronounced decrease in *Hsp70* mRNA levels after 10 days of treatment. Furthermore, in contrast to chrysin, luteolin also decreased the level of the *GstD1-GFP* reporter gene in some cases. Thus, luteolin is more biologically active than chrysin.

The results of our experiments demonstrate the ability of the flavones chrysin and luteolin to improve the lifespan of both *C. elegans* and *D. melanogaster* biological models. The possible mechanism of their action is through AMPK activation. Even though naringin demonstrated no positive effects on the lifespan of *D. melanogaster*, it produced the most pronounced effects on Nrf2 target activation. The transcriptional response data analysis of the A549 and MCF7 cell lines (resulted from the LINCS Project) revealed that rapamycin and naringin activate and inhibit some common signaling pathways. However, mostly their mechanisms of action are different.

## Author Contributions

EL, EP, MV, AZ, and AM wrote the manuscript text. EL, NZ, EP, AK, MV, EM, and SL carried out the experiments and processed the statistical analysis. AM supervised the research and the text of the manuscript. All authors read and approved the final manuscript.

## Conflict of Interest Statement

The authors declare that the research was conducted in the absence of any commercial or financial relationships that could be construed as a potential conflict of interest.

## Footnotes

1 http://www.lincsproject.org/

2 http://www.sabiosciences.com/pathwaycentral.php
