# Supplementary material for: The Evaluation of Geroprotective Effects of Selected Flavonoids in Drosophila melanogaster and Caenorhabditis elegans
Source: Front Pharmacol. 2017 Dec 7;8:884. doi: 10.3389/fphar.2017.00884 (PMC5770640; doi:10.3389/fphar.2017.00884)
Supplement: Supplementary file 1 [file Table_1.docx]

Supplementary Material

The Evaluation of Geroprotective Effects of Selected Flavonoids in *Drosophila melanogaster* and *Caenorhabditis elegans*

Ekaterina Lashmanova, Nadezhda Zemskaya, Ekaterina Proshkina, Anna Kudryavtseva, Marina Volosnikova, Elena Marusich, Sergey Leonov, Alex Zhavoronkov, Alexey Moskalev*

*** Correspondence:** Alexey Moskalev: amoskalev@list.ru

**Supplementary Table 1.** Primers for real-time RT-PCR.

| **Target** | **Forward primer** | **Reverse primer** |
| --- | --- | --- |
| *β-Tubulin* | 5’-GCAACTCCACTGCCATCC-3' | 5'-CCTGCTCCTCCTCGAACT-3' |
| *EF1a* | 5’-GCGTGGGTTTGTGATCAGTT-3’ | 5’-GATCTTCTCCTTGCCCATCC-3’ |
| *dSir2* | 5’-TCCAGGACAGTTAGCAGCAGTG-3’ | 5’- GGCTACGATTTCGCAGCTTCTC-3’ |
| *FOXO* | 5’-TAGCAGTGCCGGATGGAAGAAC-3’ | 5’-ACCCTCATAAAGCGGTTGTGCAG-3’ |
| *Gadd45* | 5’-GCAAACGCACAACCAAAC-3' | 5'-GGCCATCAGGCAGAAGAG-3' |
| *Hid* | 5’-CATCCATGGCCACATCAGTCAG-3’ | 5’-GTTTCTTCTTCTCGGCCTTCGC-3’ |
| *Hsp70* | 5’-TCCTCAGCGGAGACCAGA-3' | 5'-CACGTTCGCCCTCATACA-3' |
| *JNK* | 5’-TGATGCTGAGGAAGTGGATGCTC-3’ | 5’- TGCTCCACAGTGTGTTCCCTTTC-3’ |
| *Mei-9* | 5’-TCCTCAAGGCCTACAGCGATTC-3' | 5'-TCCAGATAAACGCGCTCTCTTTC-3' |
| *Mus210* | 5’-AGAAGACGGTGCATTTGAGATTGC-3’ | 5’-CCTCGCAAACAATGAAGCCATCG-3’ |
| *Spn-B* | 5’-AGATTGCTGCAGATGAGCAAAGCC-3’ | 5’-TTTATAACGCACGCCAGGAGAGGT-3’ |
| *Sod1* | 5’-TGCACGAGTTCGGTGACAACAC-3’ | 5’-TCCTTGCCATACGGATTGAAGTGC-3’ |
| *Sod2* | 5’-GGAGCACGCCTACTATCTGC-3’ | 5’-GTGCTTAGCAACCGAGCTTC-3’ |
| *p53* | 5’-TCACCTTAGCGTTGAGCCTTTG-3’ | 5’-TCGGAAATTCCCTTGCCCTGAG-3’ |
| *Keap1* | 5’-ATCCGGAGAACAATGAGTGG-3’ | 5’-CATCGAAGCCACCAACAAC-3’ |
| *CncC* | 5’-GAGGTGGAAATCGGAGATGA-3' | 5’-CTGCTTGTAGAGCACCTCAGC-3’ |
| *GclC* | 5’-ATGACGAGGAGAATGAGCTG-3’ | 5’-CCATGGACTGCAAATAGCTG-3’ |
| *AMPKa* | 5’-CATCAAGTTGTACCAGGTCATATCG-3’ | 5’-GTGGTCGAGCAGCAGGTTCT-3’ |
| *Cyp4e2* | 5’-CGGCAAGAATAAGTCAGA-3’ | 5’-ACGAAGTTGTCTTTTCCATACTGA-3’ |
| *Cyp6a20* | 5’-GTCCTGCCTGCACTTTCCT-3’ | 5’-ATCGAAGACCGATGCAGTTT-3’ |

**Supplementary Table 2.** Lifespan parameters of *C. elegans* after flavonoids treatment.

| **Replicates** | **Concentration** | **min** | **X±Δm** | **M** | **90%** | **max** | **MRDT** | **α** | **R_0_** | **N** |
| --- | --- | --- | --- | --- | --- | --- | --- | --- | --- | --- |
| **Naringin** | | | | | | | | | | |
| **1** | **Control** | 8 | 23.1±1.2 | 22 | 39 | 54 | 11.2 | 0.06 | 0.0141 | 84 |
|  | **0.5 μM** | 6 | 24.5±1.4 | 24 | 36 | 47 | 8.3 | 0.08 | 0.0084 | 49 |
|  | **1 μM** | 8 | 28.1±1.5 | 28**# | 41 | 54 | 8.9 | 0.08 | 0.0065 | 52 |
|  | **5 μM** | 8 | 26.7±1.3 | 24* | 44 | 54 | 9.8 | 0.07 | 0.0085 | 79 |
|  | **10 μM** | 8 | 26.8±1.2 | 26**# | 41 | 54 | 10.1 | 0.07 | 0.0089 | 91 |
|  | **100 μM** | 8 | 25.7±0.9 | 25* | 38 | 49 | 7.1 | 0.10 | 0.0056 | 96 |
| **2** | **Control** | 9 | 25.0±0.9 | 26 | 33 | 50 | 6.8 | 0.10 | 0.0057 | 79 |
|  | **0.5 μM** | 14 | 26.1±0.9 | 26 | 38 | 46 | 6.1 | 0.11 | 0.0040 | 75 |
|  | **1 μM** | 9 | 29.0±0.8 | 28**## | 41° | 50 | 6.2 | 0.11 | 0.0029 | 104 |
|  | **5 μM** | 9 | 28.1±0.9 | 28*# | 40 | 50 | 6.7 | 0.10 | 0.0038 | 109 |
|  | **10 μM** | 9 | 27.2±0.8 | 28 | 36 | 50 | 5.9 | 0.12 | 0.0032 | 98 |
| **3** | **Control** | 7 | 26.7±1.0 | 26 | 40 | 43 | 6.4 | 0.11 | 0.0039 | 97 |
|  | **0.5 μM** | 10 | 29.4±0.9 | 30 | 42 | 53 | 6.8 | 0.10 | 0.0034 | 94 |
|  | **1 μM** | 7 | 27.8±1.0 | 30 | 40 | 49 | 6.8 | 0.10 | 0.0041 | 93 |
|  | **5 μM** | 10 | 29.4±1.0 | 30# | 43 | 49 | 6.9 | 0.10 | 0.0035 | 98 |
|  | **10 μM** | 10 | 29.4±1.0 | 26# | 44° | 53 | 7.6 | 0.09 | 0.0042 | 97 |
|  | **100 μM** | 7 | 26.5±1.1 | 26 | 40 | 49 | 7.9 | 0.09 | 0.0062 | 91 |
| **Luteolin** | | | | | | | | | | |
| **1** | **Control** | 9 | 26.1±0.9 | 26 | 37 | 46 | 5.9 | 0.12 | 0.0036 | 88 |
|  | **0.5 μM** | 9 | 24.5±1.0 | 26 | 34 | 39 | 5.1 | 0.14 | 0.0031 | 58 |
|  | **1 μM** | 12 | 26.6±0.8 | 26 | 39 | 48 | 6.2 | 0.11 | 0.0039 | 86 |
|  | **5 μM** | 9 | 25.9±0.8 | 26 | 36 | 50 | 6.6 | 0.11 | 0.0048 | 101 |
|  | **10 μM** | 14 | 28.8±1.2 | 26# | 41 | 48 | 6.6 | 0.11 | 0.0034 | 54 |
| **2** | **Control** | 12 | 30.9±0.9 | 32 | 40 | 50 | 5.4 | 0.13 | 0.0015 | 80 |
|  | **0.5 μM** | 12 | 27.3±1.3 | 27** | 39 | 54 | 9.0 | 0.08 | 0.0073 | 61 |
|  | **1 μM** | 6 | 28.8±1.0 | 28 | 40 | 54 | 7.6 | 0.09 | 0.0046 | 90 |
|  | **5 μM** | 6 | 29.4±1.1 | 32 | 39 | 56 | 7.3 | 0.10 | 0.0040 | 73 |
|  | **10 μM** | 12 | 27.5±1.3 | 27 | 41 | 52 | 8.2 | 0.09 | 0.0060 | 62 |
|  | **100 μM** | 6 | 33.5±1.2 | 34## | 50°°° | 56 | 7.9 | 0.09 | 0.0030 | 89 |
| **3** | **Control** | 5 | 27.4±1.2 | 30 | 42 | 49 | 7.8 | 0.09 | 0.0054 | 75 |
|  | **0.5 μM** | 10 | 28.8±1.0 | 30 | 42 | 49 | 6.7 | 0.10 | 0.0033 | 110 |
|  | **1 μM** | 7 | 27.7±1.0 | 30 | 40 | 55 | 8.8 | 0.08 | 0.0065 | 109 |
|  | **5 μM** | 7 | 27.3±1.1 | 28 | 40 | 51 | 8.5 | 0.08 | 0.0064 | 102 |
|  | **10 μM** | 10 | 28.5±1.0 | 30 | 42 | 49 | 6.7 | 0.10 | 0.0035 | 95 |
|  | **100 μM** | 7 | 32.7±1.2 | 32**## | 49°° | 55 | 8.1 | 0.09 | 0.0034 | 90 |
| **Chrysin** | | | | | | | | | | |
| **1** | **Control** | 6 | 28.3±1.4 | 26 | 43 | 56 | 9.3 | 0.07 | 0.0068 | 67 |
|  | **0.5 μM** | 6 | 29.5±1.2 | 30 | 45 | 56 | 8.5 | 0.08 | 0.0053 | 88 |
|  | **1 μM** | 6 | 29.8±1.2 | 30 | 43 | 54 | 7.4 | 0.09 | 0.0038 | 79 |
|  | **5 μM** | 8 | 32.4±1.5 | 34*# | 47 | 54 | 8.5 | 0.08 | 0.0038 | 71 |
|  | **10 μM** | 6 | 32.6±2.0 | 34*# | 47 | 54 | 8.7 | 0.08 | 0.0039 | 41 |
|  | **100 μM** | 8 | 21.1±1.1 | 20***# | 32° | 47 | 8.4 | 0.08 | 0.0124 | 60 |
| **2** | **Control** | 5 | 27.3±1.5 | 30 | 40 | 49 | 7.5 | 0.09 | 0.0051 | 49 |
|  | **0.5 μM** | 10 | 30.7±1.2 | 30 | 44 | 53 | 7.2 | 0.10 | 0.0033 | 79 |
|  | **1 μM** | 7 | 30.9±1.2 | 32# | 44 | 55 | 8.1 | 0.09 | 0.0041 | 87 |
|  | **5 μM** | 7 | 30.0±1.1 | 30 | 44 | 53 | 8.2 | 0.09 | 0.0046 | 97 |
|  | **10 μM** | 10 | 31.7±1.1 | 32*# | 44 | 51 | 6.2 | 0.11 | 0.0020 | 78 |
|  | **100 μM** | 5 | 26.7±1.2 | 30 | 40 | 55 | 8.5 | 0.08 | 0.0068 | 82 |
| **3** | **Control** | 12 | 29.3±1.2 | 32 | 40 | 46 | 6.0 | 0.12 | 0.0024 | 58 |
|  | **0.5 μM** | 12 | 31.6±1.0 | 32 | 46 | 54 | 7.0 | 0.10 | 0.0029 | 84 |
|  | **1 μM** | 12 | 30.9±1.1 | 32 | 41 | 50 | 6.1 | 0.11 | 0.0021 | 72 |
|  | **5 μM** | 12 | 29.6±0.9 | 29 | 39 | 52 | 6.5 | 0.11 | 0.0029 | 95 |
|  | **10 μM** | 12 | 30.4±0.9 | 30.5 | 45 | 56 | 6.9 | 0.10 | 0.0032 | 104 |
|  | **100 μM** | 12 | 29.7±1.2 | 29 | 46 | 50 | 7.5 | 0.09 | 0.0040 | 69 |

M — median lifespan (days); Х±Δm — mean lifespan and standard error of mean (days); 90% — age of 90% mortality (days); min and max — minimum and maximum lifespan (days); MRDT — mortality rate time of doubling; α and R_0_ — Gompertz equation parameters; N — number of flies in a sample; * — р<0.05, ** — р<0.01, *** — р<0.001 (Gehan-Breslow-Wilcoxon test); # — р<0.05, ## — р<0.01, ### — р<0.001 (Cox-Mantel test); ° — р<0.05, °° — р<0.01, °°° — р<0.001 (Wang-Allison method).

**Supplementary Table 3.** Lifespan parameters of *aak2 C. elegans* mutant strain after flavonoids treatment.

| **Replicates** | **Concentration** | **min** | **X±Δm** | **M** | **90%** | **max** | **MRDT** | **α** | **R_0_** | **N** |
| --- | --- | --- | --- | --- | --- | --- | --- | --- | --- | --- |
| **Naringin** | | | | | | | | | | |
| **1** | **Control** | 10 | 15.5±0.9 | 14 | 25 | 31 | 5.84 | 0.11877 | 0.01547 | 51 |
|  | **5 μM** | 10 | 16.1±0.7 | 14 | 25 | 31 | 4.89 | 0.14182 | 0.01054 | 85 |
| **2** | **Control** | 7 | 18.1±0.9 | 20 | 28 | 33 | 4.84 | 0.14308 | 0.00719 | 56 |
|  | **5 μM** | 3 | 18±1 | 19 | 28 | 33 | 5.16 | 0.13426 | 0.00834 | 51 |
| **3** | **Control** | 5 | 17.5±0.8 | 17 | 26 | 31 | 4.83 | 0.14338 | 0.00799 | 64 |
|  | **5 μM** | 7 | 16.9±0.7 | 17 | 24 | 31 | 4.17 | 0.16622 | 0.0067 | 78 |
| **Luteolin** | | | | | | | | | | |
| **1** | **Control** | 10 | 15.5±0.9 | 14 | 25 | 31 | 5.84 | 0.11877 | 0.01547 | 51 |
|  | **100 μM** | 5 | 15±0.6 | 11 | 23 | 31 | 5.34 | 0.1297 | 0.01467 | 92 |
| **2** | **Control** | 7 | 18.1±0.9 | 20 | 28 | 33 | 4.84 | 0.14308 | 0.00719 | 56 |
|  | **100 μM** | 7 | 19.2±1.1 | 21 | 28 | 35 | 4.89 | 0.1417 | 0.00621 | 45 |
| **3** | **Control** | 5 | 17.5±0.8 | 17 | 26 | 31 | 4.83 | 0.14338 | 0.00799 | 64 |
|  | **100 μM** | 7 | 19.2±1 | 19 | 31 | 33 | 5.32 | 0.13036 | 0.0073 | 51 |
| **Chrysin** | | | | | | | | | | |
| **1** | **Control** | 7 | 18.1±0.9 | 20 | 28 | 33 | 4.84 | 0.14308 | 0.00719 | 56 |
|  | **10 μM** | 7 | 18.7±0.9 | 21 | 28 | 33 | 4.94 | 0.14045 | 0.0068 | 63 |
| **2** | **Control** | 5 | 17.5±0.8 | 17 | 26 | 31 | 4.83 | 0.14338 | 0.00799 | 64 |
|  | **10 μM** | 5 | 17.8±0.9 | 17 | 25 | 35 | 5.58 | 0.12421 | 0.01034 | 49 |

M — median lifespan (days); Х±Δm — mean lifespan and standard error of mean (days); 90% — age of 90% mortality (days); min and max — minimum and maximum lifespan (days); MRDT — mortality rate time of doubling; α and R_0_ — Gompertz equation parameters; N — number of flies in a sample; * — р<0.05, ** — р<0.01, *** — р<0.001 (Gehan-Breslow-Wilcoxon test); # — р<0.05, ## — р<0.01, ### — р<0.001 (Cox-Mantel test); ° — р<0.05, °° — р<0.01, °°° — р<0.001 (Wang-Allison method).

**Supplementary Table 4.** Lifespan parameters of *D. melanogaster* after naringin treatment.

| **Replicates** | **Concentration** | **min** | **X±Δm** | **M** | **90%** | **max** | **MRDT** | **α** | **R_0_** | **N** |
| --- | --- | --- | --- | --- | --- | --- | --- | --- | --- | --- |
| **Males** | | | | | | | | | | |
| **1** | **Control** | 17 | 48.6±0.9 | 46 | 61 | 73 | 6.5 | 0.11 | 0.0003 | 141 |
|  | **0.3 μM** | 20 | 47.1±1.0 | 46 | 62 | 64 | 6.9 | 0.10 | 0.0005 | 129 |
|  | **0.5 μM** | 11 | 45.5±0.9 | 46*# | 59 | 79 | 7.4 | 0.09 | 0.0008 | 144 |
|  | **1.0 μM** | 4 | 42.7±1.1 | 43***## | 61 | 64 | 8.2 | 0.08 | 0.0014 | 124 |
| **2** | **Control** | 19 | 45.6±0.9 | 50 | 58 | 59 | 5.9 | 0.12 | 0.0003 | 138 |
|  | **0.3 μM** | 16 | 46.5±0.9 | 50 | 58 | 58 | 5.6 | 0.12 | 0.0002 | 133 |
|  | **0.5 μM** | 12 | 44.5±0.9 | 46# | 57 | 58 | 5.7 | 0.12 | 0.0003 | 138 |
|  | **1.0 μM** | 4 | 42.4±0.9 | 40* | 57 | 59 | 6.8 | 0.10 | 0.0008 | 141 |
| **3** | **Control** | 6 | 59.6±0.9 | 59 | 70 | 86 | 7.0 | 0.10 | 0.0002 | 150 |
|  | **0.3 μM** | 6 | 60.8±0.8 | 63 | 70 | 91 | 6.2 | 0.11 | 0.0000 | 156 |
|  | **0.5 μM** | 10 | 61.7±0.7 | 63** | 70 | 80 | 4.3 | 0.16 | 0.0000 | 165 |
|  | **1.0 μM** | 8 | 57.9±0.9 | 62 | 69° | 70 | 4.9 | 0.14 | 0.0000 | 153 |
| **Females** | | | | | | | | | | |
| **1** | **Control** | 5 | 55.3±1.0 | 58 | 64 | 85 | 6.7 | 0.10 | 0.0002 | 135 |
|  | **0.3 μM** | 2 | 56.1±1.3 | 62*## | 74°° | 84 | 8.6 | 0.08 | 0.0000 | 137 |
|  | **0.5 μM** | 5 | 57.4±1.2 | 61## | 75°° | 85 | 8.8 | 0.08 | 0.0000 | 129 |
|  | **1.0 μM** | 9 | 59.8±1.3 | 62***### | 78°°° | 85 | 8.6 | 0.08 | 0.0004 | 140 |
| **2** | **Control** | 4 | 54.0±1.1 | 57 | 64 | 80 | 6.3 | 0.11 | 0.0002 | 142 |
|  | **0.3 μM** | 5 | 53.4±0.8 | 54 | 64 | 72 | 5.3 | 0.13 | 0.0000 | 141 |
|  | **0.5 μM** | 8 | 52.7±0.9 | 53.5**### | 62 | 79 | 6.4 | 0.11 | 0.0002 | 150 |
|  | **1.0 μM** | 3 | 53.7±1.0 | 57 | 65 | 79 | 6.7 | 0.10 | 0.0002 | 138 |
| **3** | **Control** | 6 | 60.6±1.3 | 63 | 77 | 87 | 8.1 | 0.09 | 0.0003 | 141 |
|  | **0.3 μM** | 6 | 64.3±1.3 | 66*# | 80 | 91 | 8.0 | 0.09 | 0.0002 | 149 |
|  | **0.5 μM** | 6 | 65.0±1.4 | 69***### | 80° | 90 | 7.9 | 0.09 | 0.0002 | 148 |
|  | **1.0 μM** | 6 | 62.9±1.1 | 64.5 | 77 | 84 | 7.1 | 0.10 | 0.0001 | 150 |

M — median lifespan (days); Х±Δm — mean lifespan and standard error of mean (days); 90% — age of 90% mortality (days); min and max — minimum and maximum lifespan (days); MRDT — mortality rate time of doubling; α and R_0_ — Gompertz equation parameters; N — number of flies in a sample; * — р<0.05, ** — р<0.01, *** — р<0.001 (Gehan-Breslow-Wilcoxon test); # — р<0.05, ## — р<0.01, ### — р<0.001 (Cox-Mantel test); ° — р<0.05, °° — р<0.01, °°° — р<0.001 (Wang-Allison method).

**Supplementary Table 5.** Lifespan parameters of *D. melanogaster* after luteolin treatment.

| **Replicates** | **Concentration** | **min** | **X±Δm** | **M** | **90%** | **max** | **MRDT** | **α** | **R_0_** | **N** |
| --- | --- | --- | --- | --- | --- | --- | --- | --- | --- | --- |
| **Males** | | | | | | | | | | |
| **1** | **Control** | 17 | 48.6±0.9 | 46 | 61 | 73 | 6.5 | 0.11 | 0.0003 | 141 |
|  | **0.3 μM** | 5 | 48.2±1.2 | 46 | 64°° | 81 | 10.5 | 0.07 | 0.0018 | 143 |
|  | **0.5 μM** | 2 | 50.9±1.2 | 54**### | 64 | 64 | 6.4 | 0.11 | 0.0002 | 129 |
|  | **1.0 μM** | 11 | 46.5±1.3 | 50 | 62 | 64 | 8.7 | 0.08 | 0.0011 | 145 |
| **2** | **Control** | 19 | 45.6±0.9 | 50 | 58 | 59 | 5.9 | 0.12 | 0.0003 | 138 |
|  | **0.3 μM** | 16 | 43.1±1.1 | 44## | 57 | 57 | 6.7 | 0.10 | 0.0007 | 122 |
|  | **0.5 μM** | 8 | 44.1±1.0 | 40 | 58 | 59 | 6.8 | 0.10 | 0.0007 | 120 |
|  | **1.0 μM** | 8 | 42.1±0.9 | 40* | 58° | 59 | 6.9 | 0.10 | 0.0009 | 144 |
| **3** | **Control** | 6 | 59.6±0.9 | 59 | 70 | 86 | 7.0 | 0.10 | 0.0002 | 150 |
|  | **0.3 μM** | 14 | 59.5±0.9 | 62 | 70°° | 80 | 5.5 | 0.13 | 0.0000 | 139 |
|  | **0.5 μM** | 10 | 59.2±0.9 | 62 | 70 | 84 | 6.6 | 0.11 | 0.0001 | 155 |
|  | **1.0 μM** | 28 | 58.1±0.6 | 62## | 65°°° | 72 | 3.9 | 0.18 | 0.0000 | 143 |
| **Females** | | | | | | | | | | |
| **1** | **Control** | 5 | 55.3±1.0 | 58 | 64 | 85 | 6.7 | 0.10 | 0.0002 | 135 |
|  | **0.3 μM** | 12 | 60.1±0.9 | 61***### | 70°° | 83 | 5.9 | 0.12 | 0.0000 | 135 |
|  | **0.5 μM** | 5 | 59.7±1.0 | 61***### | 71° | 81 | 5.9 | 0.12 | 0.0000 | 133 |
|  | **1.0 μM** | 9 | 59.5±0.9 | 61***### | 73° | 81 | 6.2 | 0.11 | 0.0000 | 142 |
| **2** | **Control** | 4 | 54.0±1.1 | 57 | 64 | 80 | 6.3 | 0.11 | 0.0002 | 142 |
|  | **0.3 μM** | 5 | 57.4±0.9 | 59**## | 68°° | 79 | 6.7 | 0.10 | 0.0002 | 149 |
|  | **0.5 μM** | 8 | 59.1±1.0 | 59***### | 75°° | 80 | 7.0 | 0.10 | 0.0002 | 141 |
|  | **1.0 μM** | 8 | 56.0±0.9 | 59*# | 65° | 80 | 5.7 | 0.12 | 0.0000 | 147 |
| **3** | **Control** | 6 | 60.6±1.3 | 63 | 77 | 87 | 8.1 | 0.09 | 0.0003 | 141 |
|  | **0.3 μM** | 6 | 61.2±1.2 | 64 | 73 | 83 | 6.5 | 0.11 | 0.0000 | 149 |
|  | **0.5 μM** | 6 | 61.4±1.2 | 64 | 73 | 83 | 6.6 | 0.11 | 0.0000 | 137 |
|  | **1.0 μM** | 23 | 62.5±0.9 | 68 | 70° | 86 | 5.6 | 0.12 | 0.0000 | 133 |

M — median lifespan (days); Х±Δm — mean lifespan and standard error of mean (days); 90% — age of 90% mortality (days); min and max — minimum and maximum lifespan (days); MRDT — mortality rate time of doubling; α and R_0_ — Gompertz equation parameters; N — number of flies in a sample; * — р<0.05, ** — р<0.01, *** — р<0.001 (Gehan-Breslow-Wilcoxon test); # — р<0.05, ## — р<0.01, ### — р<0.001 (Cox-Mantel test); ° — р<0.05, °° — р<0.01, °°° — р<0.001 (Wang-Allison method).

**Supplementary Table 6.** Lifespan parameters of *D. melanogaster* after chrysin treatment.

| **Replicates** | **Concentration** | **min** | **X±Δm** | **M** | **90%** | **max** | **MRDT** | **α** | **R_0_** | **N** |
| --- | --- | --- | --- | --- | --- | --- | --- | --- | --- | --- |
| **Males** | | | | | | | | | | |
| **1** | **Control** | 17 | 48.6±0.9 | 46 | 61 | 73 | 6.5 | 0.11 | 0.0003 | 141 |
|  | **0.3 μM** | 8 | 46.3±1.0 | 46 | 62 | 71 | 7.6 | 0.09 | 0.0008 | 143 |
|  | **0.5 μM** | 5 | 49.5±1.0 | 48 | 62 | 70 | 6.8 | 0.10 | 0.0004 | 127 |
|  | **1.0 μM** | 28 | 54.2±0.8 | 58***### | 62 | 71 | 5.0 | 0.14 | 0.0000 | 135 |
| **2** | **Control** | 19 | 45.6±0.9 | 50 | 58 | 59 | 5.9 | 0.12 | 0.0003 | 138 |
|  | **0.3 μM** | 9 | 46.3±1.0 | 50.5 | 58 | 58 | 5.9 | 0.12 | 0.0003 | 144 |
|  | **0.5 μM** | 8 | 46.6±0.9 | 51 | 58 | 58 | 5.6 | 0.12 | 0.0002 | 148 |
|  | **1.0 μM** | 8 | 45.6±0.8 | 47 | 58° | 59 | 5.5 | 0.13 | 0.0002 | 148 |
| **3** | **Control** | 6 | 59.6±0.9 | 59 | 70 | 86 | 7.0 | 0.10 | 0.0002 | 150 |
|  | **0.3 μM** | 5 | 57.6±1.1 | 61 | 69 | 79 | 5.9 | 0.12 | 0.0000 | 124 |
|  | **0.5 μM** | 9 | 57.7±1.1 | 61 | 69 | 78 | 6.1 | 0.11 | 0.0000 | 142 |
|  | **1.0 μM** | 19 | 59.9±1.1 | 62 | 69 | 90 | 7.5 | 0.09 | 0.0002 | 115 |
| **Females** | | | | | | | | | | |
| **1** | **Control** | 5 | 55.3±1.0 | 58 | 64 | 85 | 6.7 | 0.10 | 0.0002 | 135 |
|  | **0.3 μM** | 4 | 61.1±1.0 | 62***### | 74°°° | 81 | 6.5 | 0.11 | 0.0000 | 143 |
|  | **0.5 μM** | 5 | 64.5±1.3 | 65***### | 81°°° | 85 | 7.4 | 0.09 | 0.0001 | 143 |
|  | **1.0 μM** | 3 | 65.1±1.0 | 64***### | 78°°° | 85 | 6.6 | 0.11 | 0.0000 | 142 |
| **2** | **Control** | 4 | 54.0±1.1 | 57 | 64 | 80 | 6.3 | 0.11 | 0.0002 | 142 |
|  | **0.3 μM** | 5 | 57.3±1.0 | 58*## | 71°° | 79 | 6.7 | 0.10 | 0.0002 | 143 |
|  | **0.5 μM** | 9 | 56.5±0.8 | 58 | 67 | 78 | 5.7 | 0.12 | 0.0000 | 144 |
|  | **1.0 μM** | 8 | 56.9±0.9 | 59**## | 71° | 81 | 6.7 | 0.10 | 0.0002 | 155 |
| **3** | **Control** | 6 | 60.6±1.3 | 63 | 77 | 87 | 8.1 | 0.09 | 0.0003 | 141 |
|  | **0.3 μM** | 27 | 64.2±0.9 | 67.5 | 76 | 86 | 6.1 | 0.11 | 0.0000 | 134 |
|  | **0.5 μM** | 9 | 62.7±1.2 | 65 | 77 | 89 | 7.0 | 0.10 | 0.0001 | 129 |
|  | **1.0 μM** | 7 | 61.0±1.2 | 63 | 75 | 90 | 7.4 | 0.09 | 0.0002 | 124 |

M — median lifespan (days); Х±Δm — mean lifespan and standard error of mean (days); 90% — age of 90% mortality (days); min and max — minimum and maximum lifespan (days); MRDT — mortality rate time of doubling; α and R_0_ — Gompertz equation parameters; N — number of flies in a sample; * — р<0.05, ** — р<0.01, *** — р<0.001 (Gehan-Breslow-Wilcoxon test); # — р<0.05, ## — р<0.01, ### — р<0.001 (Cox-Mantel test); ° — р<0.05, °° — р<0.01, °°° — р<0.001 (Wang-Allison method).

**Supplementary Table 7.** Pathway-based similarity between naringin and rapamycin in A549 cells.

| **Rank of naringin** | **Rapamycin concentration** | **Time of exposure to rapamycin (hours)** | **Number of commonly regulated pathways** |
| --- | --- | --- | --- |
| **Naringin (time of exposure 6 hours, concentration 10 μM)** | | | |
| **1591** | 0.120 μM | 24 | 123 |
| **3754** | 3.333 μM | 6 | 112 |
| **4745** | 10.00 μM | 24 | 109 |
| **5910** | 1.110 μM | 24 | 105 |
| **6802** | 0.370 μM | 6 | 102 |
| **7226** | 10.00 μM | 6 | 101 |
| **8240** | 0.124 μM | 6 | 97 |
| **8750** | 0.040 μM | 24 | 96 |
| **8903** | 0.002 μM | 6 | 95 |
| **9376** | 1.111 μM | 6 | 94 |
| **9705** | 0.014 μM | 6 | 92 |
| **11298** | 3.330 μM | 24 | 87 |
| **11604** | 0.005 μM | 6 | 85 |
| **11976** | 0.370 μM | 24 | 84 |
| **13393** | 0.041 μM | 6 | 76 |
| **Naringin (time of exposure 24 hours, concentration 10 μM)** | | | |
| **348** | 0.040 μM | 24 | 88 |
| **2171** | 1.110 μM | 24 | 75 |
| **4160** | 0.005 μM | 6 | 66 |
| **5497** | 0.002 μM | 6 | 61 |
| **6217** | 1.111 μM | 6 | 59 |
| **6516** | 0.370 μM | 6 | 58 |
| **6635** | 0.120 μM | 24 | 58 |
| **7309** | 0.124 μM | 6 | 55 |
| **7552** | 3.330 μM | 24 | 54 |
| **7702** | 3.333 μM | 6 | 54 |
| **8375** | 0.014 μM | 6 | 52 |
| **8879** | 0.370 μM | 24 | 50 |
| **10505** | 0.041 μM | 6 | 46 |
| **12751** | 10.00 μM | 6 | 38 |
| **13799** | 10.00 μM | 24 | 33 |

**Supplementary Table 8.** Pathway-based similarity between naringin and rapamycin in MCF7 cells.

| **Rank of naringin** | **Rapamycin concentration** | **Time of exposure to rapamycin (hours)** | **Number of commonly regulated pathways** |
| --- | --- | --- | --- |
| **Naringin (time of exposure 6 hours, concentration 10 μM)** | | | |
| **1065** | 0.120 μM | 24 | 141 |
| **1293** | 1.110 μM | 24 | 139 |
| **1806** | 0.370 μM | 24 | 135 |
| **2516** | 0.002 μM | 24 | 131 |
| **2664** | 0.041 μM | 24 | 130 |
| **4057** | 1.111 μM | 24 | 124 |
| **4843** | 0.124 μM | 6 | 121 |
| **5977** | 0.124 μM | 24 | 118 |
| **7284** | 0.005 μM | 24 | 113 |
| **8019** | 3.330 μM | 24 | 111 |
| **8070** | 0.041 μM | 6 | 111 |
| **8273** | 0.040 μM | 24 | 110 |
| **11784** | 1.111 μM | 6 | 100 |
| **Naringin (time of exposure 24 hours, concentration 10 μM)** | | | |
| **1306** | 1.111 μM | 24 | 229 |
| **2008** | 0.041 μM | 24 | 219 |
| **2199** | 10.00 μM | 24 | 216 |
| **2461** | 0.005 μM | 6 | 213 |
| **2644** | 0.005 μM | 24 | 211 |
| **3083** | 0.002 μM | 24 | 207 |
| **3167** | 0.040 μM | 24 | 206 |
| **3425** | 0.124 μM | 24 | 203 |
| **3844** | 1.110 μM | 24 | 200 |
| **5765** | 0.120 μM | 24 | 185 |
| **6132** | 0.370 μM | 6 | 183 |
| **6905** | 0.370 μM | 24 | 178 |
| **7232** | 10.00 μM | 6 | 176 |
| **7240** | 3.330 μM | 24 | 176 |
| **7253** | 3.333 μM | 24 | 175 |
| **7746** | 0.014 μM | 24 | 172 |
| **8386** | 0.041 μM | 6 | 169 |
| **11326** | 0.014 μM | 6 | 154 |
